# Advances in imaging and treatment of atherosclerosis based on organic nanoparticles

**DOI:** 10.1063/5.0127835

**Published:** 2022-12-05

**Authors:** Shuangshuang Tu, Wenming He, Jinru Han, Aiguo Wu, Wenzhi Ren

**Affiliations:** 1 Department of Cardiology, The Affiliated Hospital of Medical School, Ningbo University, 247 Renmin Road, Jiangbei District, Ningbo, Zhejiang Province 315020, China; 2 Cixi Institute of Biomedical Engineering, International Cooperation Base of Biomedical Materials Technology and Application, Chinese Academy of Science (CAS), Key Laboratory of Magnetic Materials and Devices & Zhejiang Engineering Research Center for Biomedical Materials, Ningbo Institute of Materials Technology and Engineering, CAS, 1219 ZhongGuan West Road, Ningbo 315201, China; 3 University of Chinese Academy of Sciences, No. 1 Yanqihu East Road, Huairou District, Beijing 101408, China; 4 Advanced Energy Science and Technology Guangdong Laboratory, Huizhou 516000, China

## Abstract

Atherosclerosis, a systemic chronic inflammatory disease, can lead to thrombosis and vascular occlusion, thereby inducing a series of serious vascular diseases. Currently, distinguishing unstable plaques early and achieving more effective treatment are the two main clinical concerns in atherosclerosis. Organic nanoparticles have great potential in atherosclerotic imaging and treatment, showing superior biocompatibility, drug-loading capacity, and synthesis. This article illustrates the process of atherosclerosis onset and the key targeted cells, then systematically summarizes recent progress made in organic nanoparticle-based imaging of different types of targeted cells and therapeutic methods for atherosclerosis, including optical and acoustic-induced therapy, drug delivery, gene therapy, and immunotherapy. Finally, we discuss the major impediments that need to be addressed in future clinical practice. We believe this article will help readers to develop a comprehensive and in-depth understanding of organic nanoparticle-based atherosclerotic imaging and treatment, thus advancing further development of anti-atherosclerosis therapies.

## I. INTRODUCTION

Cardiovascular diseases remain the primary cause of global morbidity and mortality. According to the latest report from the American Heart Association, about 19 × 10^6^ deaths were attributed to cardiovascular diseases globally in 2020.^1^ Atherosclerosis, the leading cause of cardiovascular disease, is a systemic chronic inflammatory disease characterized by the formation of plaques within the arterial wall. Plaques are constructed by the build-up of lipid and cellular components under the inner wall of the blood vessel. Generally, the growth or rupture of atherosclerotic plaques will result in thrombosis and vascular occlusion, which can lead to severe outcomes such as acute myocardial infraction, stroke, and peripheral artery disease.^2^ Therefore, early detection and effective treatment of atherosclerosis are essential to delay plaque growth and prevent life-threatening events.

Currently, the common clinical diagnosis techniques for atherosclerosis, such as ultrasound imaging (US), magnetic resonance imaging (MRI), and computed tomography (CT), can only provide information at the plaque structure and anatomical levels but are difficult to accurately identify vulnerable plaques in the early stage.^3^ Clinical treatments for atherosclerosis include drug therapies and surgical interventions. Common medication prescriptions for atherosclerosis patients include lipid-lowering drugs, glucose-lowering drugs, antihypertensive drugs, and antiplatelet and anticoagulant drugs.^4^ Invasive treatments, such as stent installation and coronary artery bypass grafting, are widely used to treat severe atherosclerotic lesions or advanced plaques. However, although these treatments for atherosclerosis have certain positive effects, they may inevitably facilitate corresponding side effects and complications, leading to coronary heart disease with high morbidity and mortality rates. Consequently, given the current situation of diagnosis and treatment for atherosclerosis, it is urgent to explore new imaging and treatment strategies to identify early vulnerable plaques and to specifically enhance the therapeutic effect of anti-atherosclerosis. This is a process where challenges and opportunities coexist.

In recent years, benefiting from the rapid development of nanotechnology and nanomedicine, the investigation of nanoparticles (NPs) for anti-atherosclerosis purposes has become a research hotspot. Currently, NPs are mainly designed for imaging and treatment of atherosclerosis targets, which can be key cells and cytokines in the development of plaques, such as intercellular adhesion molecule-1 (ICAM-1) and vascular cell adhesion molecule-1 (VCAM-1) in activated endothelial cells^5^ and smooth muscle cells,^6^ macrophages,^7^ and foam cells in inflammatory plaques.^8^ NPs can be designed as either imaging agents or carriers of imaging agents for use in the diagnosis of atherosclerosis. For example, Fe_3_O_4_ NPs can specifically enhance the MRI contrast of atherosclerotic plaques to increase the rate of detection.^9^ NPs can also achieve efficient treatment, acting as therapeutic agents or drug carriers to mediate photothermal therapy (PTT),^10^ photodynamic therapy (PDT),^11^ sonodynamic therapy (SDT),^12^ drug delivery,^13^ and so on.

In general, NPs applied for anti-atherosclerosis purposes may be divided primarily into inorganic NPs and organic NPs. Compared to inorganic NPs, organic NPs, such as polymeric NPs,^14^ liposomes,^15^ micelles,^16^ and high-density lipoprotein NPs,^17^ have the advantages of high drug-loading capacity, good biocompatibility, long circulation time, and rapid elimination from the body. Organic NPs are modified to optimize their target specificity, tissue-penetration depth, duration, and so on, providing the possibility for early diagnosis and effective treatment of atherosclerosis. More importantly, the U.S. Food and Drug Administration has approved several kinds of liposome- and micelle-based medicines for clinic cancer treatment, which has inspired scientists to explore the potential clinical transformation of organic NPs for anti-atherosclerosis purposes.

This article illustrates the development process of atherosclerosis, especially the primary cells involved, and then summarizes organic NP-based dual-modality imaging of these cells and therapeutic approaches for atherosclerosis, including optical and acoustic-excited therapy, drug delivery, gene therapy, and immunotherapy. Finally, this article discusses the opportunities and challenges of organic NPs in the clinical translation of anti-atherosclerosis in future.

## II. THE OCCURRENCE AND DEVELOPMENT OF ATHEROSCLEROSIS

**FIG. 1.**
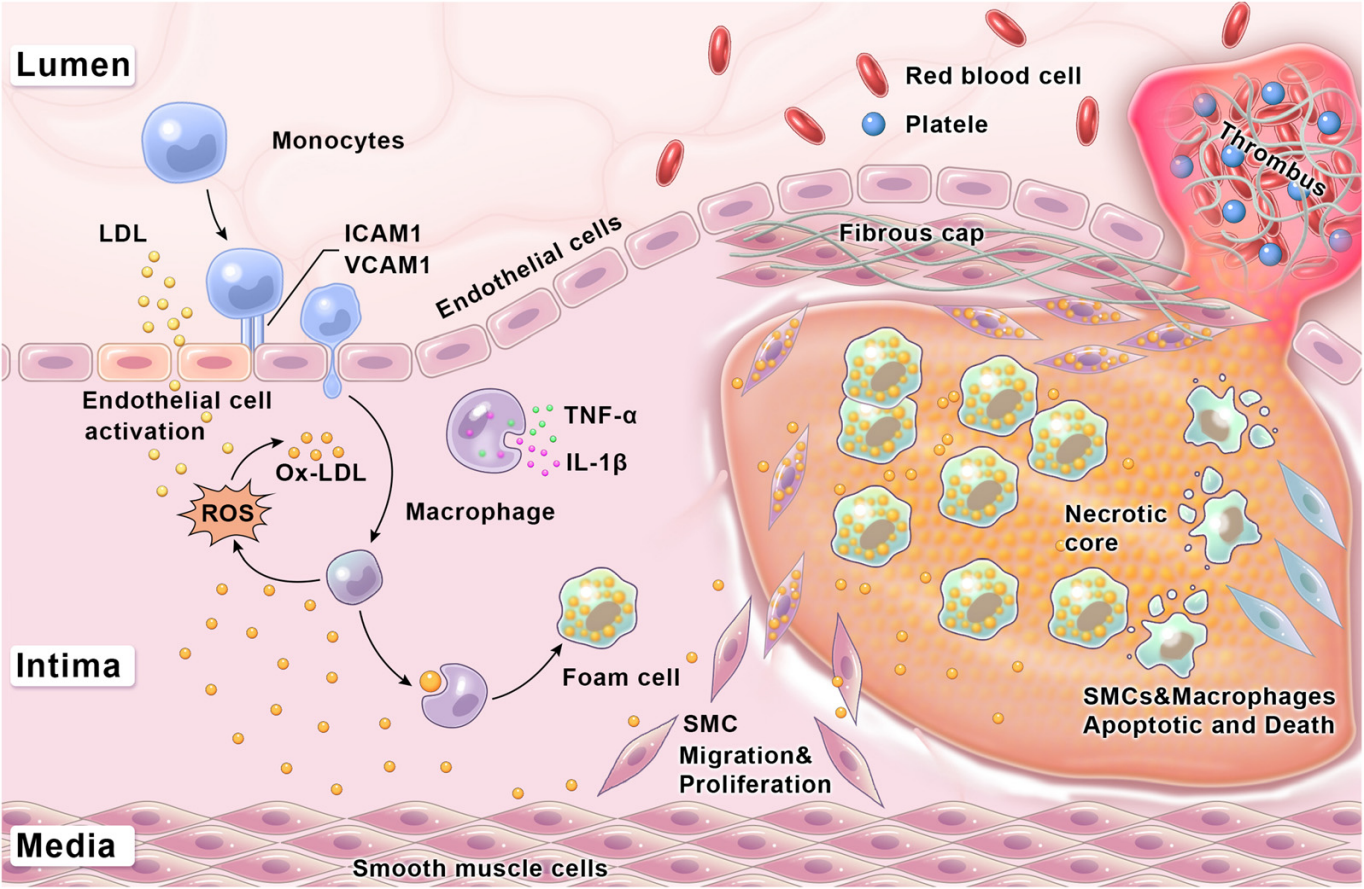
Schematic illustration of the atherosclerosis progression.

Atherosclerosis is characterized by the formation of plaques underneath the lining of blood vessels, which consist of lipids, inflammatory cells, and fibrous deposits. The development and progression of atherosclerosis is a complex and constantly changing process,^2,5–7,18^ which can be mainly divided into three stages as follows (Fig. 1).

In the first stage, vascular endothelial cells are activated under harmful stimuli, leading to endothelial dysfunction. The activated endothelial cells secrete a variety of chemokines and inflammatory cell molecules, which promote the migration and recruitment of monocytes in the vascular intima. Meanwhile, low-density lipoproteins (LDLs) are modified into oxidized LDLs (ox-LDLs) and accumulate in the inner blood vessel wall. Moreover, endothelial cells express leukocyte-adhesion molecules, such as ICAM-1 and VCAM-1, promoting the adhesion of monocytes to the blood vessel wall.^18^ Monocytes are later activated by macrophage colony-stimulating factor and differentiate into macrophages. In addition, the monocyte-derived macrophages can be further differentiated into foam cells through the uptake of ox-LDLs.^5,6^

In the second stage, the pro-inflammatory cytokines secreted by lymphocytes and inflammatory cells promote the migration of vascular smooth muscle cells into intima and drive them to shift from a contractile phenotype to a synthetic phenotype. Then, vascular smooth muscle cells proliferate and secrete extracellular matrix components, such as collagen and proteoglycan, to form a fibrous cap. Moreover, vascular smooth muscle cells can form foam cells by ingesting ox-LDLs.^2,5,6^

The last stage of atherosclerosis is the most severe, encompassing plaque rupture and thrombosis. The thinning of the fibrous cap and the formation of a necrotic core are important factors that cause plaque rupture. Inflammatory macrophages can induce smooth muscle cell apoptosis and reduce collagen synthesis. Moreover, inflammatory macrophages and synthetic phenotypic vascular smooth muscle cells can produce matrix metalloproteinases (MMPs) such as MMP-2 and MMP-9, which can cause collagen degradation, fibrous cap thinning, and even plaque rupture.^2,5^ In addition, pro-inflammatory cytokines, such as interleukin (IL)-1β and tumor necrosis factor (TNF)-α, can further induce foam cell necrosis, enlarge the necrotic core, and increase the risks of plaque instability and rupture. Plaque rupture can result in thrombosis and vascular occlusion, which eventually leads to a series of severe cardiovascular events such as myocardial infarction, stroke, and even death.^7^

Figure 1 clearly shows the key process of atherosclerotic plaque development, which involves five types of main cells, specifically endothelial cells, vascular smooth muscle cells, monocytes, macrophages, and foam cells. Activated endothelial cells can recruit and promote monocytes to convert into macrophages. The uptake of ox-LDL can differentiate macrophages into foam cells. Moreover, the accumulation of death foam cells can further drive the formation of plaque necrotic cores and exacerbate the inflammatory response. In addition, vascular smooth muscle cells can evolve into foam cells and fibrous caps, and the phenotypic transformation of vascular smooth muscle cells affects the stability and outcome of plaques. Therefore, endothelial cells, vascular smooth muscle cells, monocytes, macrophages, and foam cells can be considered potential targets for imaging and treatment of atherosclerosis.

## III. ORGANIC NPs FOR ATHEROSCLEROTIC IMAGING

### A. Single-mode imaging

At present, clinical imaging techniques still have limitations in accurate distinguishing early vulnerable and stable plaques. As shown in Table I, each imaging modality has its own unique benefits and drawbacks, including temporal and spatial resolution, sensitivity, tissue-penetration depth, and duration. Therefore, it is possible to compare plaques information using various imaging modalities, such as MRI, near-infrared fluorescence imaging (NIRFI), photoacoustic imaging (PAI), positron emission tomography (PET), CT, and US, by studying the application of organic NPs in atherosclerosis-targeted imaging.

**TABLE I.** Single-mode imaging modalities and applications of organic NPs.

Image	Organic NPs	Energy form	Imaging agents	Advantages	Disadvantages	References
MRI	Polymeric NPs liposomes micelles	Radiofrequency waves	Fe^3+^ polymeric NP, gadolinium (Gd)-containing liposomes, Gd/superparamagnetic iron oxide (SPIO)-containing liposomes, Gd-containing micelles, iron oxide/manganese oxide micelles	High spatial resolution, deep tissue penetration, high soft-tissue contrast	Low sensitivity, high cost, long scan time	19–23
NIRFI	Polymeric NPs liposomes micelles	Near-infrared light	Boron dipyrromethene fluorophore (BOD)-L-βGal polymeric NPs, indocyanine green (ICG)-containing liposomes, ICG-containing micelles	High sensitivity, short scan time, deep tissue penetration	Low spatial resolution, poor target location	24–26
PAI	Polymeric NPs	Photo-induced US waves	Semiconducting polymeric NPs	High sensitivity, high spatial resolution, short scan time	Limited tissue-penetration depth	27
PET	Polymeric NPs	Annihilation photos	**^64^**Cu-labeled polymeric NPs, **^89^**Zr-labeled polymeric NPs	High sensitivity, high specificity, quantitative	High cost, low spatial resolution, lack of anatomical reference	28–30
CT	Liposomes	X-rays	Iodixanol-containing liposomes	Short scan time, high density resolution, high lesion detection rate	Radiation, low spatial resolution, low sensitivity	31
US	Polymeric NPs	Ultrasound waves	Perfluorooctyl bromide (PFOB) NPs, perfluoropentane (PFP) NPs	Simple, real-time imaging, low cost	Limited tissue-penetration depth, low resolution	32, 33

### B. Dual-mode imaging

In recent years, a new imaging mode formed by the fusion of multiple ones has attracted widespread attention. This multi-mode imaging can overcome the limitations of single-mode imaging and enhance the imaging effect by combining the advantages of various imaging modalities to realize early diagnosis of atherosclerosis. To demonstrate the potential of multimodal imaging, we mainly summarize the latest development of organic NP-based dual-modality imaging of atherosclerosis (Table II). Among them, we will introduce the imaging advances classified according to target cell types in atherosclerosis.

**TABLE II.** Dual-mode imaging modalities and applications of organic NPs.

Image	Organic NPs	Imaging agents	Cell/molecular targeting	Animal model	Imaging effect	References
MRI-NIRFI	Micelles polymeric NPs	Gd/-Cy7, Fe**_2_**O**_3_**/DyLight 800	Monocytes, fibrin, platelet integrin αIIbβ3	ApoE**^−/−^** mice	Notable MRI signal and fluorescence signal enhancements in the aorta	22, 34, 35
PET-MRI	Polymeric NPs	^18^F/Gd	Macrophages	ApoE**^−/−^** mice	Increased PET signal and MRI in the aortic arches	36
MRI-US	Polymeric NPs	SPIO/PFOB, Fe**_3_**O**_4_**/PFP	Intraplaque neovascularization, macrophages	Aorta balloon-injury (ABI) rats, ApoE**^−/−^** mice	A broader detectable time-window, a longer duration of T_2_W signal attenuation, and a hyperechoic signal detected in the aorta of targeted group	33, 37
US-NIRFI	Polymeric NPs	PFOB/Cy5.5	Foam cells	ApoE**^−/−^** mice	Strong US signal and fluorescence signal in the aorta of targeted group	32
PET-CT	Polymeric NPs	**^64^**Cu	Macrophages	ApoE**^−/−^** mice	Continued signal enhancement three times in aortic plaques of the targeted group after injection for 24 h	38
MRI-FI	Liposomes	Fe**_3_**O**_4_**/fluorescein isothiocyanate (FITC)	Mouse aortic endothelial cells	ApoE**^−/−^** mice	Increased fluorescence signal and MRI contrast-to-noise ratio (CNR) in the aortic arches	39

#### 1. Monocyte imaging

**FIG. 2.**
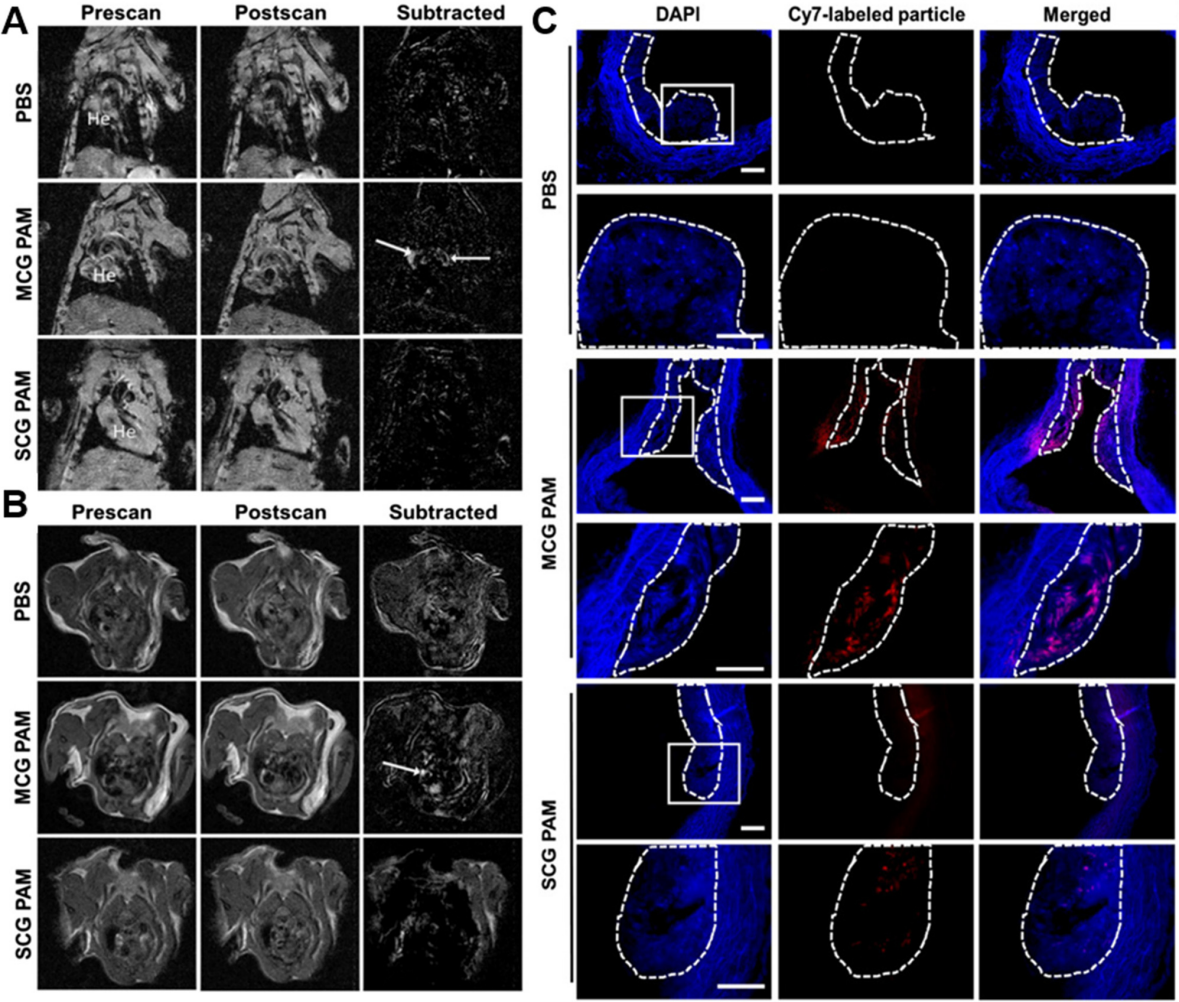
MRI scans of mice treated with phosphate buffer saline (PBS), untargeted (SCG PAM) micelles, and targeted (MCG PAM) micelles. (a) Coronal scans and (b) transverse scans, MRI signal enhancement of aortic (arrow) plaques in the MCG PAM group. (c) Fluorescence image of aortic plaques showing good plaque targeting in the MCG PAM group (red). Reproduced with permission from Chin *et al.*, Adv. Ther. **3**, 1900196 (2020). Copyright 2020 John Wiley and Sons, Clearance Center, Inc.^22^

In the early stage of atherosclerosis, activated endothelial cells secrete monocyte chemoattractant protein-1/C-C chemokine ligand 2 (MCP-1/CCL2), and its receptor C-C Motif Chemokine Receptor 2 (CCR2) is highly expressed on the surface of monocytes. MCP-1 binds and promotes monocyte migration and recruitment to the vascular endothelium.^40,41^ Subsequently, monocytes infiltrating the arterial wall are stimulated to differentiate into macrophages and further form foam cells by ingesting ox-LDL, which promotes the progression of atherosclerosis.^18^ The migration and recruitment of monocytes to the vascular endothelium is a key process in early atherosclerosis. Therefore, monocytes can be used as potential imaging targets for the diagnosis of atherosclerosis. Chung *et al.* synthesized polypeptide amphiphilic (MCG PAM) micelles by coupling MCP-1 polypeptide, collagenase inhibitor Col-1 polypeptide, MRI contrast agent Gd, and near-infrared fluorescent dye Cy7. The prepared MCG PAM micelles were applied for MRI and NIRFI dual-mode imaging of monocytes in atherosclerotic plaques. This dual-mode imaging approach combines the high spatial resolution of MRI and the high sensitivity of NIRFI. Figure 2 shows that, in the MRI scan, the aortic MRI signal of the MCG PAM group was significantly enhanced, and the signal-to-noise ratio was significantly higher than that of the control group. In addition, Fig. 2(c) shows a clear fluorescence signal in the aortic plaque area treated by MCG PAM micelles. All the results indicate that the MCG PAM micelles have good imaging ability to target atherosclerotic plaques.^22^

#### 2. Macrophage imaging

**FIG. 3.**
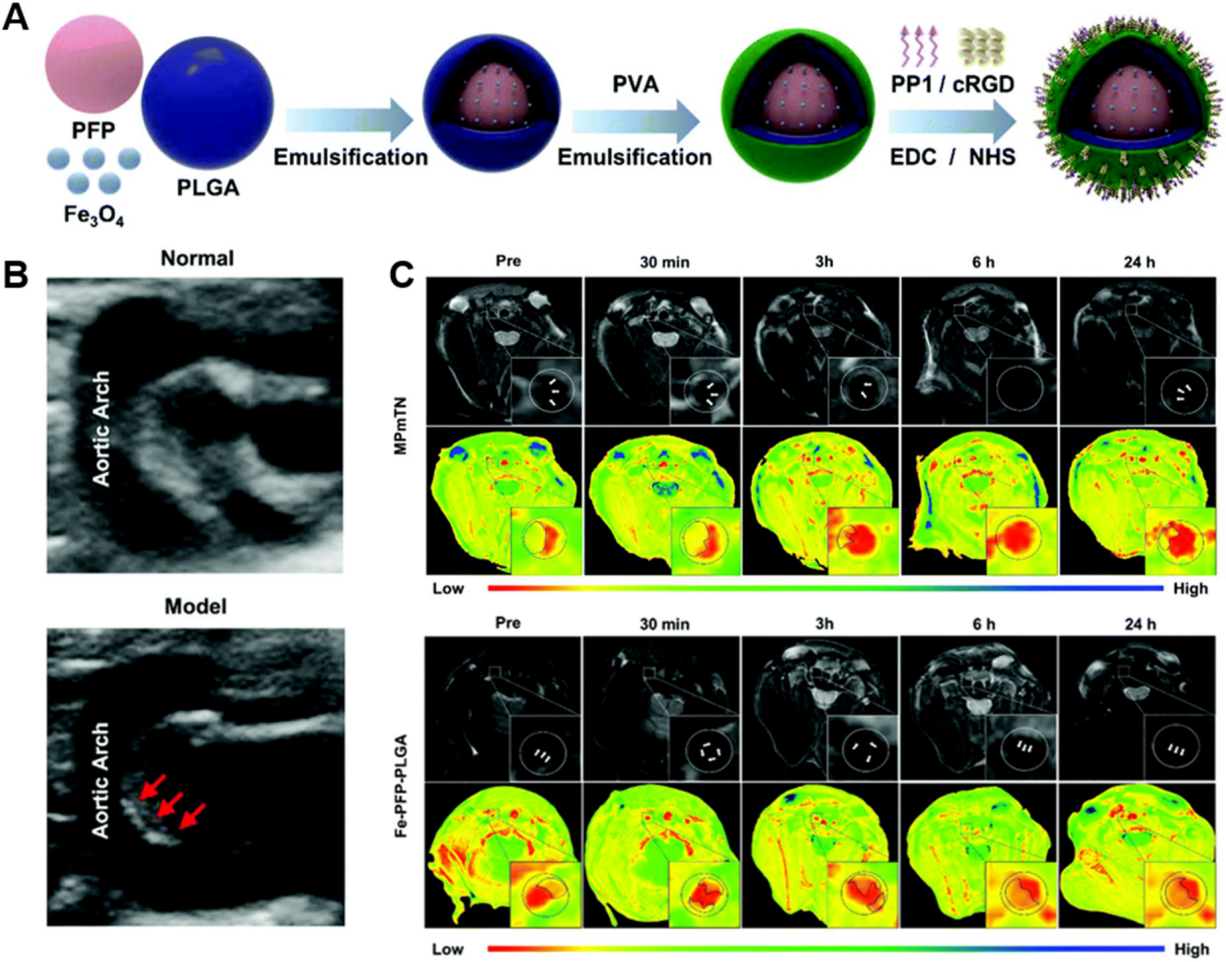
(a) Preparation scheme of MPmTN. (b) US images in aortic plaques of normal mice (left) and apoE^−/−^ mice (right). (c) MRI scans and pseudo-color images of aortic plaques after the injection of Fe–PFP–PLGA or MPmTN at different time points. Reproduced with permission from Gao *et al.*, Nanoscale **13**, 8623–8638 (2021). Copyright 2021 Royal Society of Chemistry Clearance Center, Inc.^33^

Macrophages are key cells that promote the occurrence and development of atherosclerosis. Macrophages ingest ox-LDL to transform into foam cells and secrete a series of pro-inflammatory cytokines to amplify the inflammatory response, which eventually results in necrotic core formation and plaque rupture.^7^ Due to the fact that macrophages play an important role in the instability of atherosclerotic plaques, macrophage imaging is an appealing strategy for the diagnosis of atherosclerotic plaques. Class A scavenger receptors (SR-A), scavenger receptor for ox-LDL, are highly expressed on the surface of macrophages, promoting the formation of macrophage-derived foam cells.^42,43^ Therefore, SR-A is a viable target for macrophages in atherosclerotic plaques. It was reported that PP1 polypeptide is a high-affinity ligand specific to SR-A.^44^ Gao *et al.* used poly(lactic-co-glycolic acid) (PLGA) NPs to load the contrast imaging material Fe_3_O_4_, PFP, and specific plaque-targeted peptides PP1 and cyclic RGD to prepare MPmTN. The prepared MPmTN used for MRI and US dual-mode imaging revealed the abilities of both high-resolution MRI and real-time US imaging. Figure 3 shows that MPmTN could significantly reduce the signal intensity of T_2_-weighted images. In addition, compared to the control group, the US signal intensity of the MPmTN group was significantly greater, indicating that MPmTN containing Fe_3_O_4_ and PFP facilitates good MRI and US imaging.^33^

#### 3. Foam cell imaging

**FIG. 4.**
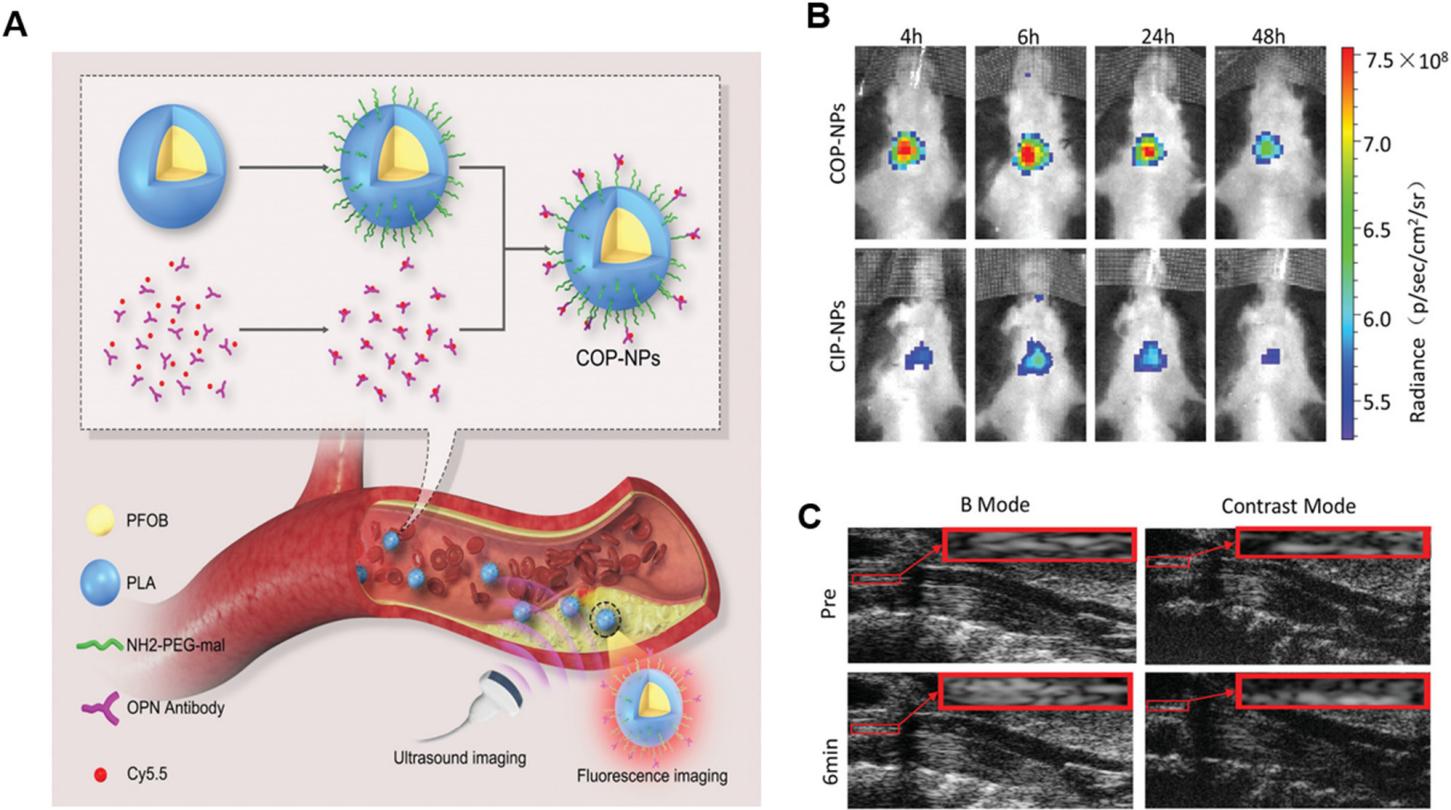
(a) The scheme of PFOB-, Cy5.5-, and OPN antibody-loaded polymeric NPs (CoP-NPs) for US and NIRF dual-mode imaging of foam cells in atherosclerotic plaques. (b) Fluorescence images of atherosclerotic mice after the injection of untargeted control group (CIP-NPs) and CoP-NPs at different time points. (c) US images of the carotid artery acquired through B and contrast modes before the injection and 6 min after the injection of CoP-NPs. Reproduced with permission from Li *et al.*, Macromol. Biosci. **20**, e1900279 (2020). Copyright 2020 Authors, licensed under a Creative Commons Attribution (CC BY) license.^32^

The formation of foam cells is a main sign of the presence of atherosclerotic lesions.^45^ Foam cells are mainly derived from macrophages with the features of increased uptake of ox-LDL, increased cholesterol esterification, and decreased cholesterol efflux, which facilitate the accumulation of excess lipid droplets. In addition, lipid-rich foam cells promote the formation of fatty streaks and plaques. Foam cell death can result in the formation of a necrotic core, which correlates with an increased risk of plaque rupture.^8,46^ Therefore, targeted foam cell imaging is of great significance for the early diagnosis of atherosclerotic plaques. In atherosclerotic lesions, osteopontin (OPN) is highly expressed in macrophages, foam cells, and synthetic phenotypic vascular smooth muscle cells.^3,47^ Based on these findings, OPN can be used as a potential molecular target in the imaging of foam cells in atherosclerosis lesions. Li *et al.* wrapped US contrast agent PFOB with PLGA, then further coupled the OPN antibody labeled with a near-infrared fluorescent dye (Cy5.5) through a surface-modified activator, to prepare US and NIRFI dual-mode imaging NPs. *In vivo* optical imaging and US imaging results of carotid artery plaques revealed that the fluorescence signal intensity of the CoP-NP group was greater than that of the control group at different time points after the injection of NPs through the tail vein. Compared to pre-injection, the CoP-NP group showed an obvious US signal 6 min after injection (Fig. 4). These results indicate that CoP-NPs have promise in realizing the visualization of atherosclerotic plaques.^32^

#### 4. Plaque neovascularization imaging

Plaque neovascularization is the main feature of vulnerable plaques.^48^ Neovascularization promotes atherosclerosis lesion progression, leading to plaque instability and rupture. Vascular endothelial growth factor receptor 2 (VEGFR-2), which is highly expressed on neovascular endothelial cells in plaques, plays an important role in neovascularization and endothelial cell proliferation.^49,50^ Therefore, VEGFR-2 is expected to be a potential molecular imaging target for plaque neovascularization. Chen *et al.* prepared a polymer nanocapsule VEGFR-2-targeted NC (VTNC), which contained the US contrast agent PFOB, magnetic resonance contrast agent SPIO, and VEGFR-2 antibody for US and MRI dual-mode imaging of atherosclerotic neovascularization. *In vitro* experiments showed that VTNCs could specifically recognize neovascular endothelial cells. In addition, the contrast of US imaging and magnetic resonance T_2_-weighted imaging in the VTNC group was significantly enhanced. *In vivo* experiments revealed that US imaging contrast of the VTNC group was also significantly enhanced, and the enhancement duration was longer, with the average and peak grayscale intensity (GSI) values being significantly higher than those of control groups. Compared to the control groups, the attenuation duration of the MRI T_2_-weighted signal in the VTNC group was longer, and the mean and peak signals of CNR were significantly higher. These results suggest that the VTNCs have a good targeted imaging ability for plaque neovascularization.^37^

## IV. ORGANIC NPs FOR ATHEROSCLEROSIS TREATMENT

Based on the characteristics of good biocompatibility, long circulation time, and easy degradation, organic NPs are widely used in the treatment of atherosclerosis. Organic NPs may be considered good carriers for loading therapeutic agents, such as photothermal agents, photosensitizers, sonosensitizers, and chemotherapeutic drugs. In this review, we mainly summarize the recent progress of organic NP-mediated therapeutic approaches for atherosclerosis, such as PTT, PDT, SDT, drug delivery, gene therapy, and immunotherapy.

### A. Optical and acoustic excited organic NP-based therapy for atherosclerosis

Due to the advantages of minimal invasiveness, no radiation pollution, high efficiency, high flexibility, and few side effects, NP-mediated phototherapy and acoustic therapy have become research hotspots in the treatment of atherosclerotic plaques. At present, phototherapy based on organic NPs mainly encompasses PTT and PDT. Meanwhile, SDT has also attracted extensive attention in the treatment of atherosclerosis. Next, we will cover organic NP-mediated PTT, PDT, and SDT for atherosclerosis.

#### 1. PTT based on organic NPs for atherosclerosis

The process of PTT is roughly divided into three steps as follows. First, a large amount of photothermal agent is delivered and accumulated in the target tissue. Then, the photothermal agent located in the target tissue is irradiated with a laser. Finally, the photothermal agent absorbs photons, then converts absorbed light energy into heat energy to generate a high local temperature, thereby causing targeted cell death. In addition, PTT can produce different therapeutic effects at different temperatures. Studies have shown that, when the temperature is 37 °C–41 °C, the physiological state of cells can be altered, for example, increase ion exchange of cell membrane and acceleration of the blood flow. When the temperature ranges from 41 °C to 48 °C, the treatment is called “thermotherapy,” which can lead to protein denaturation and temporary cell inactivation. Temperature higher than 50 °C can cause necrosis and irreversible damage to cells and tissues.^51,52^

Since biological tissues have the smallest absorption and scattering of near-infrared light, near-infrared light can penetrate more deep tissues.^53,54^ Therefore, photothermal agents are expected to have strong absorption and great photothermal conversion performance under near-infrared light. Being nontoxic to normal tissue in the dark conditions is also crucial. In addition, photothermal agents should possess excellent properties, such as easy modification and degradation. Research studies on inorganic NP-mediated PTT have been widely reported, which is attributed to the NPs' excellent photothermal conversion performance and high stability under near-infrared light.^10,55–59^ In addition, organic photothermal agents, such as fluorescent dyes,^60,61^ polypyrrole,^62^ and polyaniline NPs,^63^ with good biocompatibility and degradable properties, have been widely used in cancer treatment.^64^ However, at present, there is no report on organic NP-mediated PTT in the treatment of atherosclerosis.

#### 2. PDT based on organic NPs for atherosclerosis

PDT is an emerging minimally invasive treatment technology that can be used for the treatment of many diseases. PDT requires three basic elements as follows: (1) a light source that activates the photosensitizer, (2) a photosensitizer that can be selective to target cells or tissues, and (3) dissolved oxygen. In the general process of PDT, photosensitizers that accumulate in diseased tissues are activated by specific wavelengths of light to produce reactive oxygen species (ROS), leading to cell apoptosis, autophagy, or necrosis.

Studies have proved that PDT has great potential in the treatment of atherosclerosis. During therapy, the photosensitizer can target plaques without damaging normal blood vessel walls, which makes PDT an exceedingly attractive treatment for atherosclerosis. However, choosing the right photosensitizer is key to the success of PDT. Selective accumulation of the photosensitizer in a plaque is one of the most important factors in determining the effect of PDT. Other important features include low dark toxicity, low side effects, deep tissue penetration, and targeted activation.

Although the first-generation photosensitizer hematoporphyrin and its derivatives have been proven to obviously selectively accumulate in atherosclerotic plaques, their therapeutic efficiency are limited by the poor penetration of activated light at 630 nm wavelength.^65^ In addition, adverse reactions, such as skin phototoxicity, further limit their clinical application. Second-generation photosensitizers, such as porphyrins, chlorides, and dyes, show increased plaque selectivity and decreased skin phototoxicity. It is noted that second-generation photosensitizers have a longer absorption wavelength to realize better tissue penetration.

Allison *et al.* found that verteprofin irradiated by a 692-nm laser could selectively accumulate in rabbit atherosclerotic plaques.^66^ Peng *et al.* demonstrated that 5-aminolevulinic acid (5-ALA)-mediated PDT triggered by a 635-nm laser could significantly decrease macrophage content and effectively inhibit atherosclerotic plaque progression.^67^ Hayase *et al.* reported that motexafin lutetium (MLu) activated under a 732-nm laser could significantly reduce the content of macrophages and delay the progression of atherosclerosis without damaging the blood vessel wall.^68^ Eldar *et al.* discovered for the first time that atherosclerotic plaques in rabbits preferentially ingest water-soluble phthalocyanine dyes.^69^ However, phthalocyanine derivatives have limitations, such as slow clearance and poor water solubility, which limit their application in atherosclerotic PDT.

Third-generation photosensitizers have been actively developed to overcome the shortcomings of second-generation photosensitizers, new-generation photosensitizers are required to possess selective accumulation and sufficient concentration in plaques, thus improving the efficiency of PDT. Due to the traits of NPs, such as their large specific surface area and easy modification, they can act as excellent carriers for photosensitizer delivery to increase accumulated concentrations in atherosclerotic plaques. Considering their advantages of good biocompatibility and biodegradability, organic NPs have enormous potential as carriers for loading photosensitizers to achieve high efficiency in atherosclerotic PDT. For example, Jain *et al.* developed verteprofin-loaded liposomes for use in atherosclerosis PDT, and their results showed that, under 690-nm laser irradiation, the liposomes significantly accumulated in atherosclerotic plaques. In addition, liposome-mediated PDT could induce significant apoptosis of macrophages, reduce the vascular function of vascular smooth muscle cells, and inhibit vasoconstriction.^70^

Chlorin e6 (Ce6) as a promising second-generation photosensitizer has been widely used in cancer PDT.^71–73^ At present, several studies have reported on Ce6-mediated PDT in the treatment of atherosclerosis. For example, Kim *et al.* developed hyaluronic acid (HA) NPs loaded with Ce6, and their results showed that 670-nm laser irradiated NPs could produce enhanced photodynamic effect and induce phototoxic effect to cause macrophage death.^74^ In another report, Kałas *et al.* used liposomes to load Ce6 for atherosclerosis PDT. *In vitro* studies showed that Ce6-loaded liposomes could significantly accumulate in macrophages, and further irradiated by 655-nm laser to generate ROS to inactivate macrophages.^75^ Meta-tetra(hydroxyphenyl)chorin (mTHPC) is one of the most effective photosensitizers in PDT for cancer.^76,77^ Wennink *et al.* reported on mTHPC-loaded micelles used for atherosclerosis PDT and found that, due to the high lipase activity in macrophages, the micelles were easily degraded and the loaded mTHPC was released quickly, resulting in high phototoxicity. In subsequent experiments, efforts should be made to improve the stability of the micelles, thereby increasing their accumulation in atherosclerotic plaques and improving the efficacy of PDT.^78^ Interestingly, Spyropoulos-Antonakakis *et al.* combined the photosensitizer ZnPc with PAMAM dendrimers (G0) and found that, compared to G0 without photosensitizer, G0 with ZnPc significantly accumulated in atherosclerotic plaques.^79^ In summary, targeted increasing accumulation of photosensitizer in plaque is one of key elements to the success of PDT in atherosclerosis treatment.

Near-infrared light can penetrate deep tissues, giving NPs loaded with near-infrared photosensitizers great potential for use in atherosclerosis PDT. However, there is no report on the anti-atherosclerosis potential of organic NP-mediated PDT under near-infrared laser irradiation.

#### 3. SDT based on organic NPs for atherosclerosis

SDT, an emerging noninvasive treatment method, is derived from PDT. Like the mechanism of PDT, the sonosensitizer accumulates in the target tissue and gains energy when activated by US irradiation to exert local cytotoxicity and induce cell death. Compared to PDT, SDT achieves more sufficient deep tissue penetration and highly selective accumulation in target tissues.^80^ Therefore, SDT represents a good prospect for anti-atherosclerosis treatment. Sonosensitizers, the key to SDT, are supposed to have attractive characteristics, such as low toxicity to normal tissues, high sensitivity to US, easy removal from the body, and—most importantly—high specificity for target tissues.^12^

Studies have shown that protoporphyrin IX and 5-ALA can highly accumulate in atherosclerotic plaques,^67,81^ which can be used as potential sonosensitizers for SDT reliably. Tian's team conducted a series of studies on 5-ALA-based SDT for atherosclerosis. They found that 5-ALA-based SDT could induce THP-1 macrophage apoptosis via ROS generation and mitochondrial membrane potential (MMP) loss,^82^ induce the apoptosis of THP-1 macrophage-derived foam cells via the mitochondrial-caspase pathway,^83^ inhibit the progression of atherosclerosis and stabilize plaques by removing macrophages and suppressing matrix degradation,^84^ increase the stability of atherosclerotic plaques by inducing heme oxygenase-1 expression,^85^ and promote the polarization of Th2 cells to delay atherosclerosis progression.^86^ *In vivo* and *in vitro* experiments have demonstrated that 5-ALA-based SDT could quickly and effectively inhibit the progression of atherosclerosis, which might be related to its induction of macrophage apoptosis, exocytosis enhancement, inflammation reduction, cholesterol efflux promotion, and plaque stabilization. Tian's team further studied the efficacy of 5-ALA-based SDT in patients with atherosclerotic peripheral artery disease. There were more significant effects on plaque shrinkage and lumen enlargement using 5-ALA-based SDT compared to atorvastatin treatment for 1 week of treatment. In addition, the combinations of both treatments could inhibit the progression of atherosclerotic synergistically.^87,88^ Interestingly, it has been found that some Chinese herbal medicines have good sonodynamic activity and relative safety profiles.^89^ Tian's team reported that the Chinese herbal medicines emodin and curcumin, which mediate SDT to induce the apoptosis and necrosis of THP-1-derived macrophages, can be used as new sonosensitizers for atherosclerosis SDT to inhibit the progression of atherosclerosis.^90,91^

**FIG. 5.**
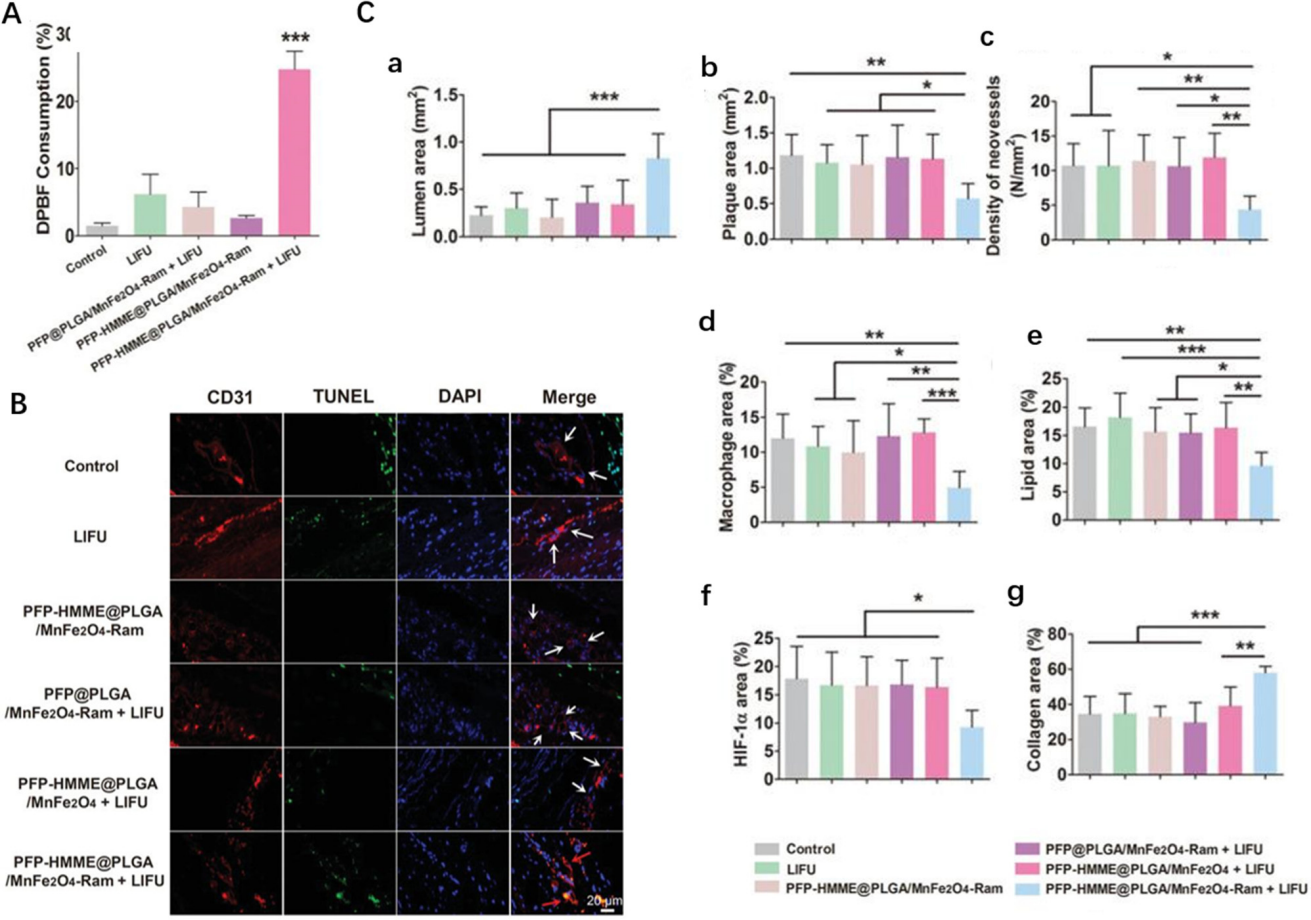
(a) The consumption of 1,3‐diphenylisobenzofuran (DPBF) upon low-intensity focused US irradiation. (b) Fluorescence images of NP-mediated SDT promoted apoptosis of aortic plaque endothelial cells and neovessels. (c) Quantitative results of histopathology (a)–(g) after 28 days of treatment. Reproduced with permission from Yao *et al.*, Adv. Sci. **8**, e2100850 (2021). Copyright 2021 Authors, licensed under a Creative Commons Attribution (CC BY) license.^93^

To identify a more effective sonosensitizer, its sufficient accumulation in plaques is essential. Organic NPs can deliver sonosensitizer to plaques efficiently given their large specific surface area, good biocompatibility, and easy modification. Hematoporphyrin monomethyl ether (HMME) is a porphyrin derivative that can be used as a safe and effective sonosensitizer to mediate SDT.^92^ Yao *et al.* prepared HMME-loaded polymer NPs for SDT of atherosclerotic plaque neovascularization, which showed good mitochondria-targeting properties in rat aortic endothelial cells (RAECs). PFP-HMME@PLGA/MnFe_2_O_4_-Ram NPs exhibited obviously higher ROS production efficacy under US stimulation [Fig. 5(a)]; compared to the control group, as shown in Fig. 5(b), PFP-HMME@PLGA/MnFe_2_O_4_-Ram NPs inhibited the proliferation, migration, and tubule formation of RAECs and induced RAEC apoptosis significantly. After advanced plaque rabbits were treated, the NP group exhibited a reduced number of neovessels (by approximately 1.35-fold) on day 3, indicating that NP-mediated SDT significantly induced plaque neovascular endothelial cell apoptosis in this context. As shown in Fig. 5(c), after 28 days of treatment, compared to in the control group, the lumen area was increased by 272.3%, plaque area was decreased by 51.8%, neovessel density was decreased by 59.1%, the macrophage content was reduced by 59%, lipid counts were reduced by 41.9%, the hypoxia-inducible factor-1α concentration was reduced by 48%, and the collagen content was increased by 67.4% in the NP group, respectively, which indicated that NP-mediated SDT significantly inhibited plaque neovascularization and stabilized atherosclerotic plaques.^93^

Interestingly, SDT can mediate different cell death pathways according to the US intensity. Sun *et al.* studied the relationship between US intensity and the proportion of apoptotic cells using real-time ROS and mitochondrial membrane potential (MMP) detection and found that US at low intensity (<0.48 W/cm^2^) had little effect on ROS and MMP concentrations in macrophages. When the US intensity was increased from 0.48 to 0.84 W/cm^2^, however, the rate of macrophage apoptosis increased. Continuing to increase the US intensity decreased the cell apoptosis rate and increased the cell necrosis rate.^94^ Significantly, *in vivo* study reported that the US intensity of 1.5 W/cm^2^ induced the highest percentage of plaque macrophage apoptosis in the SDT groups with different US intensities.^84^ These results suggest that the efficacy of SDT-induced macrophage apoptosis is controllable to a certain extent. Standardization of US parameters could maximize the proportion of macrophage apoptosis in plaques, thus improving the effect of SDT in the treatment of atherosclerosis.

In summary, the efficacy of SDT depends on many factors, such as the type and dosage of sonosensitizers and the US parameters. In addition, it is of great significance to choose a suitable carrier for SDT.

### B. Drug delivery based on organic NPs for atherosclerosis

At present, clinical drugs available for the treatment of atherosclerosis mainly include lipid-lowering, anticoagulant and antiplatelet, blood pressure- and blood sugar-controlling, and anti-inflammatory medications. However, the therapeutic effect of these drugs is largely unsatisfactory due to their insufficient accumulation in plaques, which further leads to undesirable side effects. To improve the therapeutic effect and reduce the side effects of treatments, researchers are committed to developing various NPs capable of drug delivery for the treatment of atherosclerosis. Polymers, liposomes, and micelles, which have good biocompatibility and biodegradability, have been considered as main anti-atherosclerotic drug carriers. As shown in Table III, selecting suitable carriers and therapeutic drugs, and improving their specific targeting of plaques are key challenges to overcome for successful treatment of atherosclerosis.

**TABLE III.** Organic NPs for drug delivery in atherosclerosis.

Organic NPs	Drug	Cell/molecular targeting	Animal model	Therapeutic effect
Polymeric NPs	Rapamycin, atorvastatin, methotrexate, pioglitazone, LXR agonist GW3965	Human umbilical vein vessel endothelial cells (HUVECs), RAW264.7 cells, macrophages, monocytes and macrophages, CD68+ macrophages	ApoE**^−/−^** mice, ApoE**^−/−^** mice, ApoE**^−/−^** mice, ApoE**^−/−^** mice, LDLR**^−/−^** mice	The average area ratio of plaque to the vascular lumen was reduced by 11.77%, 5.68%, and 0.72% in the aorta root, aortic arch, and abdominal aorta, respectively.^95^ TNF-α, IL-1β, IL-1α, and ICAM-1 levels were significantly reduced, and the plaque area was reduced by 69%.^96^ TNF-α and IL-6 levels were significantly reduced, and the plaque burden was reduced by 50%.^97^ Significantly decreased the number of inflammatory monocytes and buried fibrous caps, increased the fibrous cap thickness, and inhibited atherosclerotic plaque rupture.^98^ The secretion of MCP-1 and TNF-α was significantly suppressed, and the CD^68+^ area was reduced by 50%.^99^
Liposomes	Quercetin, LXR agonist GW3965, pioglitazone, atorvastatin calcium and curcumin	Macrophages, foam cells, endothelial cells and M1 macrophages, endothelial cells	ApoE**^−/−^** mice, LDLR**^−/−^** mice, ApoE**^−/−^** mice, ApoE**^−/−^** mice	Decreased the area of aortic lesion to 8.6%.^106^ The macrophage content was significantly decreased (twofold). The collagen percentage in plaques was significantly increased (threefold)^16^. Slightly reduced the atherosclerotic plaque area in the aortic root and remarkably increased the content of collagen in plaques.^107^ The percentage of the thoracic aortic plaque area was decreased to 17.14%.^108^
Micelles	Rapamycin, simvastatin, berberine, prednisolone, celastrol, andrographolide	HUVECs and RAW264.7 cells, RAW264.7 cells, macrophages, RAW264.7 cells, RAW264.7 cells, RAW264.7 cells	ApoE**^−/−^** mice, ApoE**^−/−^** mice, ApoE**^−/−^** mice, ApoE**^−/−^** mice, LDLR**^−/−^** mice, ApoE**^−/−^** mice	Reduced the aortic plaque area to 5.67% and 4.89%.^111^ Significantly reduced the lesion area.^112^ Significantly inhibited cholesteryl content in the aortic artery.^113^ Significantly reduced the atherosclerotic plaque area in the aortic arch and decreased the lipid-rich necrotic core area.^114^ The plaque area was significantly decreased.^115^ Significantly reduced the atherosclerotic plaque area in the aortic root.^116^

Currently, the most-studied polymeric NPs for anti-atherosclerosis are hyaluronic acid (HA)-based and PLGA-based NPs.^95–99^ Recent studies have shown that HA NPs can actively target atherosclerotic plaques.^100^ Hossaini Nasr *et al.* assessed the anti-atherosclerosis effect of HA NPs loaded with the clinical drug atorvastatin and found that, compared to in the free atorvastatin group, the group with HA-NPs loaded with atorvastatin experienced a better anti-inflammatory effect *in vivo* and *in vitro* after 1 week of treatment.^96^ Interestingly, some researchers have reported that HA NPs can also effectively treat atherosclerosis by loading drugs that promote the polarization of M2 macrophages.^101,102^ Same as HA NPs, PLGA NPs are also widely used in the treatment of atherosclerosis. Zhang *et al.* prepared liver X receptor (LXR) agonist GW3965 loaded PLGA NPs. Their results showed that, 2 weeks after injection, the number of CD68-positive macrophages in the plaques of low-density lipoprotein (LDL) receptor-deficient (LDLR**^−/−^**) mice were reduced by 50%, which effectively inhibited the progression of atherosclerosis. Moreover, mice in the NPs group did not experience liver steatosis as a side effect.^99^ In another study, Nakashiro *et al.* reported that PLGA NPs loaded with peroxisome proliferator-activated receptor-γ agonist pioglitazone (PIO) could induce M2 macrophage polarization and up-regulate anti-inflammatory cytokines to reduce the inflammatory response and delay atherosclerosis progression.^98^ In addition, PLGA NPs loaded with the anti-inflammatory cytokine interleukin (IL)-10 also showed satisfied anti-atherosclerosis effects.^103^

Given their special physical and chemical properties, liposomes have been widely applied as drug-delivery systems in cancer therapy.^104,105^ In recent years, delivery of anti-atherosclerosis drugs using liposomes has also attracted widespread attention.^16,106–108^ Considering that curcumin can reduce the side effects of statins, Li *et al.* designed liposomes loaded with atorvastatin and curcumin (AC-Lipo) for combination therapy of atherosclerosis. They found that, compared to liposomes loaded with only atorvastatin, AC-Lipo significantly reduced the expression of ICAM-1 and E-selectin on plaque endothelial cells, leading to decreased atherosclerotic plaque content, showing AC-Lipo could synergistically reduce plaque lipid content and inflammatory cytokine expression.^108^ In addition to endothelial cells, macrophages could become important targets for drug delivery therapy of atherosclerosis. At present, many studies have reported that liposomes are used to deliver drugs to plaque macrophages, showing good anti-atherosclerosis effects. For example, Fang *et al.* studied the targeted therapy for atherosclerosis by delivering telmisartan with folate-modified liposomes. They found that the liposomes showed good targeting ability of macrophages and were significantly aggregated in atherosclerotic plaques. After 12 weeks of treatment, the liposomes significantly inhibited advanced atherosclerotic plaque lesions, including facilitating plaque regression and stabilization, which may be related to the decreased macrophage content, increased cholesterol efflux, and increased collagen content.^109^ In another report, Benne *et al.* loaded LXR agonist GW3965 in liposomes and functionalized them with Lyp-1 (CGNKRTRGC) to target the p32 receptor, which is highly expressed on plaque foam cells. Their results showed that targeted liposomes exhibited preferential uptake by foam cells *in vitro* and were more significantly accumulated in atherosclerotic plaques in mice compared to non-targeted liposomes. Targeted liposomes also showed increased retention in plaque macrophages and reduced the content of macrophages by 50% without LXR agonist side effects.^16^ In addition, liposomes targeting macrophages could effectively reduce plaque inflammation by delivering the anti-inflammatory cytokine IL-10.^110^

**FIG. 6.**
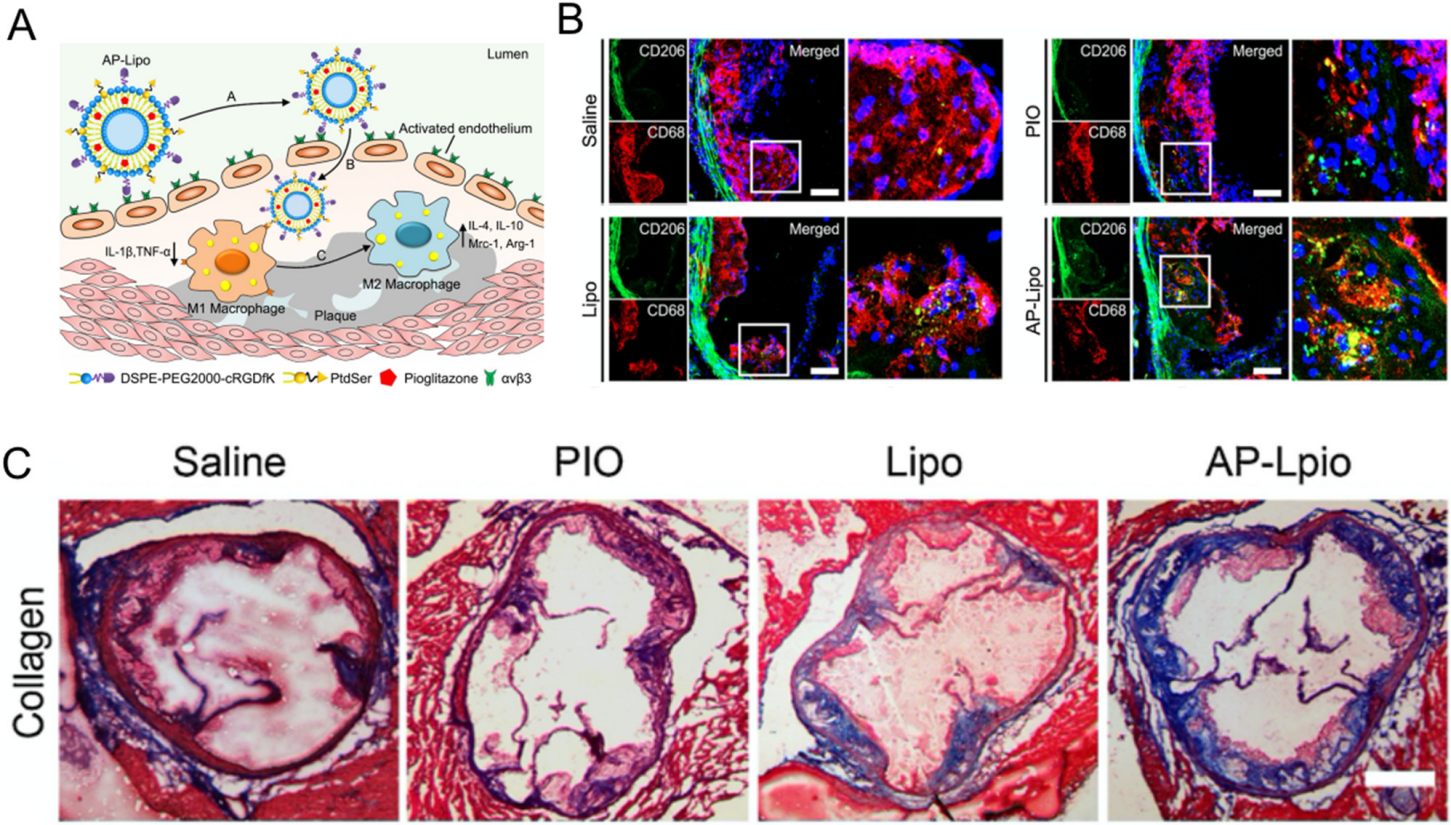
(a) The scheme of AP-Lipo for atherosclerosis therapy. (b) Confocal microscopy images of M2 macrophages with CD68 (red) and CD206 (green) immunostaining. (c) Masson's trichrome staining of aortic plaques after different treatments. Reproduced with permission from Wu *et al.*, J. Controlled Release **316**, 236–249 (2019). Copyright 2019 Elsevier.^107^

Interestingly, to improve the efficiency of drug delivery, Zhang *et al.* prepared apoptotic body biomimic liposomes (AP-Lipo). Phosphatidylserine was used to modify liposomes to send out “phagocytosis” signals, which can be effectively recognized and internalized by M1 macrophages. The therapeutic drug PIO promoted the polarization of M2 macrophages to produce anti-inflammatory effects. Cyclo (Arg-Gly-Asp-D-Tyr-Cys) (cRGDfK) peptides can specifically bind to αvβ3 integrin expressed on vascular endothelial cells in atherosclerotic plaques. As shown in Fig. 6(b), M2 macrophages were detected by anti-CD206 antibody, and the results revealed that, compared to the control group, the AP-Lipo group exhibited the strongest green fluorescence in plaques, indicating that AP-Lipo could significantly induce the polarization of M2 macrophages and increase the number of M2 macrophages in plaques. Masson's trichrome staining also showed that the AP-Lipo group had significantly increased collagen content in plaques, which enhances their stability, compared to the control group [Fig. 6(c)]. In summary, the innovative apoptotic body biomimetic liposome represents a potential drug-delivery system for the treatment of atherosclerosis.^107^

**FIG. 7.**
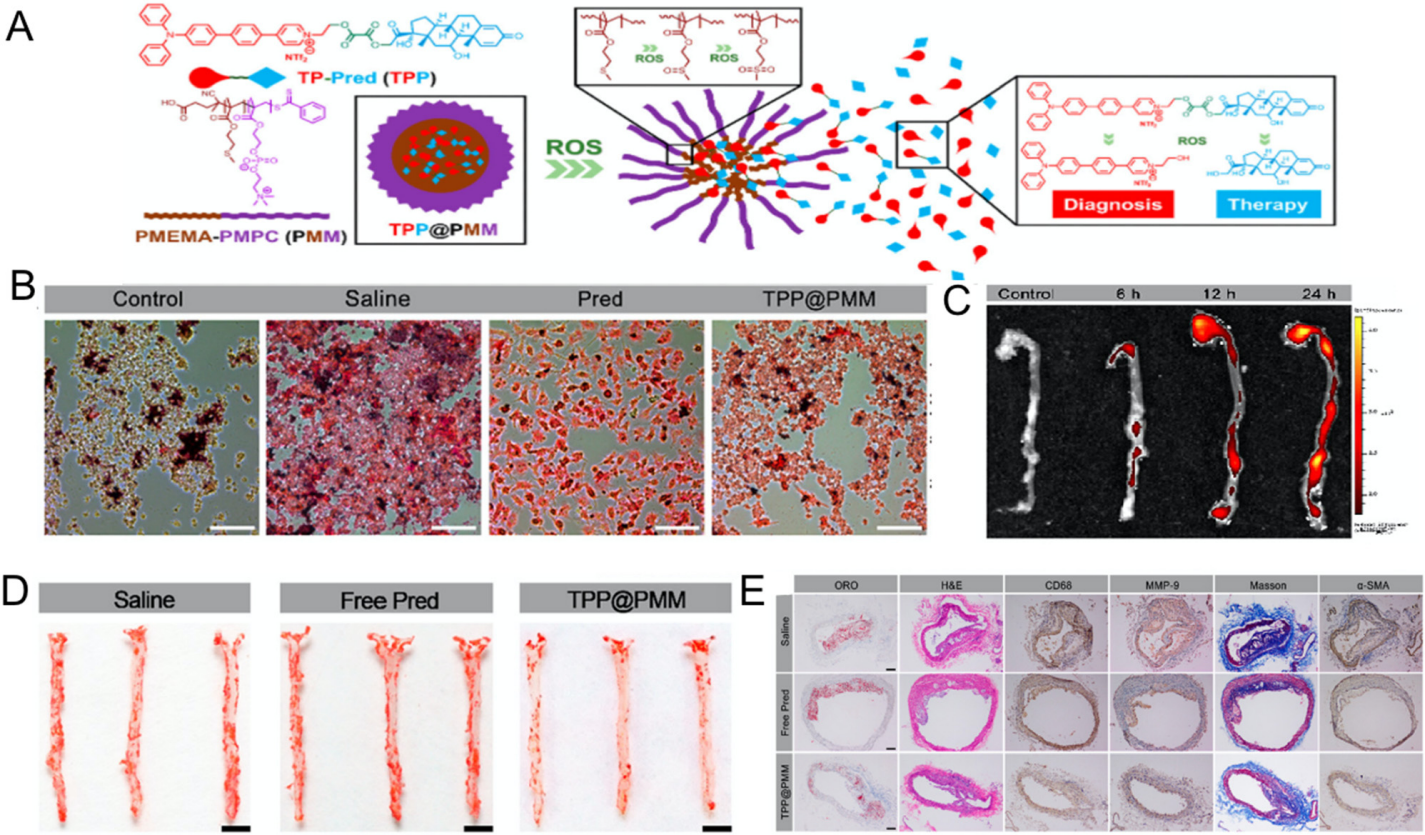
(a) Preparation scheme of TPP@PMM. (b) Optical microscopy images of foam cells with different treatments. (c) *In vitro* fluorescence images of TPP@PMM in mouse aortas. (d) Oil red O-stained images of mice aorta with different treatments. (e) Pathological staining of the aortic arch in different treatment groups. Reproduced with permission from Ma *et al.*, ACS Nano **14**, 5862–5873 (2020). Copyright 2020 American Chemical Society.^114^

Similar to liposomes, micelles loaded with anti-inflammatory drugs have shown satisfactory effects for the treatment of atherosclerosis.^111–116^ Remarkably, Wang's team prepared an innovative and attractive micelle for anti-atherosclerosis, connecting the therapeutic drug prednisolone to a two-photon fluorophore via a ROS-sensitive bond to form the compound TPP, which was then loaded by the amphiphilic polymer poly(2-methylthio ethanol methacrylate) (PMEMA)-PMPC (PMM) to form ROS-responsive micelles (TPP@PMM) through self-assembly. As shown in Fig. 7(b), the TPP@PMM group reduced uptake of ox-LDL by RAW264.7 cells and decreased formation of foam cells. TPP@PMM accumulated rapidly in the aortas of atherosclerotic mice for 6 h after injection and was stored up within 24 h [Fig. 7(c)]. As shown in Fig. 7(d), the TPP@PMM group had significantly inhibited plaque formation compared to the control group. Histochemical analysis of plaques demonstrated that, compared to the control group, the mice treated with TPP@PMM also showed fewer necrotic cores, less macrophage activation, and reduced inflammatory cytokine expression [Fig. 7(e)]. The most fascinating conclusion of this study is that TPP@PMM can realize diagnostic imaging and therapeutic intervention at the same time.^114^

### C. Gene therapy based on organic NPs for atherosclerosis

Gene therapy has been considered a promising treatment for atherosclerosis. Studies have shown that organic NPs loaded with small interfering RNA (siRNA) can induce the inhibition of specific genes expression, thereby possessing anti-atherosclerosis effects. Zhao *et al.* prepared a polymer NPs loaded with lectin-like ox-LDL receptor 1 (LOX-1) siRNA and modified with HA for the gene therapy of atherosclerosis. Compared to free LOX-1 siRNA, the NPs loaded with LOX-1 had a better macrophage-targeting ability, which could reduce the plaque area and decrease lipid and macrophage contents, showing a significant anti-atherosclerotic effect. In addition, NPs coated with higher-molecular-weight HA and higher-coating-density HA showed better anti-atherosclerosis efficacy. Therefore, polymeric-based siRNA NPs can be considered a class of promising gene-delivery systems for the gene therapy of atherosclerosis.^117^ In another report, Tao *et al.* synthesized siRNA NPs targeting the *Camk2g* gene, which inhibited atherosclerosis progression and increased plaque stability by suppressing the expression of CaMKIIγ in macrophages.^118^ Interestingly, Hou *et al.* found that dexamethasone together with mTOR gene inhibition could be significantly anti-inflammatory. CaP/siNC/HA-dexamethasone hemisuccinate (DSH) particles remarkably reduced the IL-1β, IL-6, and TNF-α messenger RNA expression.^119^ Moreover, Majmudar *et al.* prepared anti-inflammatory liposomes, which delivered CCR2 siRNA to monocytes to inhibit CCR2 expression, thereby preventing an inflammatory response in plaques. Their results showed that atherosclerotic plaque area and monocyte content were significantly reduced by treatment.^120^ In summary, organic NPs are promising carriers for delivering siRNA to atherosclerotic plaques, which will lay a foundation for expansion and improvement of gene therapy of atherosclerosis.

### D. Immunotherapy based on organic NPs for atherosclerosis

Recently, immunotherapy has shown great potential in preventing and suppressing atherosclerosis. Immune cells involved in anti-atherosclerosis include regulatory T-cells (Tregs), follicular helper T-cells, and B-cells. These cells, activated by specific antigens, will inhibit the progression of atherosclerosis by releasing anti-inflammatory cytokines or antibodies. In addition, carriers loaded with atherosclerosis-related antigens or selective adjuvants can induce immune responses in body, which can mobilize Tregs, follicular helper T-cell, and B-cell responses.^121^ Therefore, a successful vaccination strategy is essential to improve the immunotherapy effect of atherosclerosis, and the use of organic NP-based vaccines has become an attractive vaccination strategy. According to reports, apoptotic cells can induce B-cells to secrete the anti-inflammatory cytokine IL-10.^122^ Immunoglobulin M produced by B1a-cells can induce the production of anti-inflammatory cytokines to inhibit atherosclerosis.^123,124^ Hosseini *et al.* reported that phosphatidylserine liposomes can mimic apoptotic cells to target B1a-cell and produce polyreactive immunoglobulin M antibodies to reduce inflammation in plaques.^125^ In addition, Benne *et al.* found that Anionic 1,2-distearoyl-sn-glycero-3-phosphoglycerol (DSPG) liposomes, encapsulating an LDL-derived peptide antigen, can significantly induce the proliferation of Tregs and reduce the area of atherosclerotic plaques.^126^ In order to improve the efficacy of a cholesteryl ester transfer protein (CETP) vaccine, Aghebati *et al.* prepared liposomes (Lipo-CETP) encapsulating tetanus toxoid-CETP (TT-CETP) and found that, compared to the control group, the Lipo-CETP group had significantly increased anti-TT-CETP-specific antibodies in serum and expression levels of interferon-γ and IL-4 in monocytes, suggesting good effects for atherosclerosis protection.^127^ In another study, the nanoliposome immunogenic fused PCSK9-tetanus (IFPT) vaccine, prepared by Momtazi-Borojeni *et al.*, also showed good anti-atherosclerotic effect.^128^ The above-mentioned studies confirmed that liposomes may have great potential as vaccine-adjuvant delivery systems for immunotherapy of atherosclerosis. In addition to liposomes, micelles have also been reported to mediate vaccine treatment of atherosclerosis. In 2019, Gutiérrez-Vidal *et al.* prepared a micelle-encapsulating vaccine, HB-ATV-8, for the immunotherapy of porcine atherosclerosis, and their results showed that, compared to the control group, the vaccine group had significant higher concentration of anti-CETP immunoglobulin G; furthermore, 7 months after vaccine administration, the level of plasma triglyceride was significantly reduced and the expression of atherosclerosis-related genes had largely returned to normal. As such, micelles-based vaccines may achieve effective prevention and inhibition of atherosclerosis.^129^

## V. CONCLUSION AND PERSPECTIVES

Atherosclerosis is a major cause of cardiovascular diseases that leads to high morbidity and mortality rates worldwide. Therefore, early diagnosis and efficient treatment of atherosclerosis are essential to delay lesions and prevent life-threatening events. With the rapid development of nanomedical technology, researchers have prepared various NPs for the imaging and treatment of atherosclerosis.

Compared to inorganic NPs, organic NPs—including polymer NPs, liposomes, micelles, and high-density lipoprotein NPs—have advantages of high drug loading ability, good biocompatibility, long circulation time, easy metabolism, rapid removal from the body, and other characteristics, suggesting their great potential in anti-atherosclerosis efforts.

Each imaging modality has its own unique advantages and disadvantages, including sensitivity, temporal and spatial resolution, and tissue-penetration depth and duration. Single-mode imaging is not enough to provide accurate imaging information. Therefore, considering the advantages of dual-mode imaging and the mainly involved cells in atherosclerosis, this article focuses on the studies of dual-mode imaging of these major abnormal cells, such as MRI-NIRFI, MRI-US, and US-NIRFI of monocytes, macrophages, foam cells, neovascular endothelial cells, and so on. This article could be helpful for forming a more systematic and comprehensive understanding of atherosclerosis imaging.

In the area of atherosclerosis treatment, this article summarized the progress of work involving organic NP-based optical and acoustic excitation therapy, drug delivery, gene therapy, and immunotherapy. Organic NPs have shown great potential to serve as successful carriers for imaging and therapeutic agents by targeting atherosclerotic plaques. However, even though some animal experiments have proved that organic NPs carry the advantages of accurate diagnosis and efficient treatment, further reliable research and supporting data are still needed to propel them into clinical application. This is both a major challenge and a potential opportunity.

## Data Availability

Data sharing is not applicable to this article as no new data were created or analyzed in this study.
